# Manufacturing and Characterization of 3D Miniature Polymer Lattice Structures Using Fused Filament Fabrication

**DOI:** 10.3390/polym13040635

**Published:** 2021-02-20

**Authors:** Rafael Guerra Silva, María Josefina Torres, Jorge Zahr Viñuela, Arístides González Zamora

**Affiliations:** 1 School of Mechanical Engineering, Pontificia Universidad Católica de Valparaíso, Av. Los Carrera 01567, Quilpué 2430000, Chile; josefina.torres@pucv.cl (M.J.T.); jorge.zahr@pucv.cl (J.Z.V.); 2 School of Mechanical Engineering, Universidad Central de Venezuela, Los Chaguaramos 1050, Caracas, Venezuela; aristides.gonzalez@gmail.com

**Keywords:** lattice, additive manufacturing, fused filament fabrication, manufacturability

## Abstract

The potential of additive manufacturing to produce architected lattice structures is remarkable, but restrictions imposed by manufacturing processes lead to practical limits on the form and dimension of structures that can be produced. In the present work, the capabilities of fused filament fabrication (FFF) to produce miniature lattices were explored, as they represent an inexpensive option for the production of polymer custom-made lattice structures. First, fused filament fabrication design guidelines were tested to assess their validity for miniature unit cells and lattice structures. The predictions were contrasted with the results of printing tests, showing some discrepancies between expected outcomes and resulting printed structures. It was possible to print functional 3D miniature open cell polymer lattice structures without support, even when some FFF guidelines were infringed, i.e., recommended minimum strut thickness and maximum overhang angle. Hence, a broad range of lattice structures with complex topologies are possible, beyond the cubic-type cell arrangements. Nevertheless, there are hard limits in 3D printing of miniature lattice structures. Strut thickness, length and orientation were identified as critical parameters in miniature lattice structures. Printed lattices that did not fully comply with FFF guidelines were capable of bearing compressive loads, even if surface quality and accuracy issues could not be fully resolved. Nevertheless, 3D printed FFF lattice structures could represent an improvement compared to other additive manufacturing processes, as they offer good control of cell geometry, and does not require additional post-processing.

## 1. Introduction

Although cellular materials have been used in different engineering applications over the years [1], additive manufacturing (AM) opens up new opportunities for their design and development. While in conventional cellular materials cell size can only be partially controlled, AM offers control over cell size and shape, shape and size of struts, topology of the structure, and many other features [2]. These features provide new opportunities for the design and fabrication of biomedical devices, such as tissue scaffolds [3,4,5,6], artificial high porosity structures with complex geometries designed to support tissue growth, for instance bone [3,7]. AM lattices could be used for that purpose, but there is a need for characterizing their accuracy and mechanical performance to fully assess their suitability for the manufacturing of scaffolds [3]. Extensive mechanical experiments and modeling are necessary due to the unique behaviors of the different designs [8].

Recently, special attention has been given to the development of design and optimization methods for lattice structures [9,10,11,12,13], most of them based on analytical or numerical methods. However, while analytical and numerical models of lattice structures could be used to assess their mechanical behavior, they usually do not consider local geometrical irregularities, an unavoidable consequence of the manufacturing process [14]. Furthermore, it does not provide information regarding the manufacturability of lattice structures via additive manufacturing. The quality of the lattice structure, including dimensional accuracy, surface quality, residual stresses, microstructure, and overall variability does influence its performance [14,15].

Lattices are also considered an excellent alternative for producing lightweight, strong parts using AM [16]. There has been some interest in assessing the capabilities and limits of AM for printing lattice structures, i.e., cellular materials, although in a non-methodical fashion. Mueller et al. [17] identified some restrictions of the laser sintering process, similar to those reported for general AM: overhangs should not exceed 45° and only open cell structures are possible, so the non-molten metal powder could be removed. However, limitations such as the minimum cell size, thickness of walls/struts, or cell geometry were not evaluated.

Zhou [18] reported on the manufacturability of selected miniature lattice structures using different technologies that included fused filament fabrication (FFF), selective laser sintering (SLS), stereolithography (SLA) and direct light processing (DLP), evidencing that FFF does not have an adequate resolution to print microstructures, in opposition to SLS and DLP printers. In FFF, broken struts and uneven thickness were evident. Minimal strut thickness ranged from 0.2 in SL and DLP to 2 mm in FFF. The periodic lattice structures were modeled using the software nTopology (nTopology Inc., New York, USA). Neither dimensions nor geometric features of the tested lattices were reported, although the STL files of the models are available online [19].

FFF could seem inadequate when compared to stereolithography and selective laser sintering technologies for the production of 3D miniature lattice structures, as the process has limited accuracy, manufacturing of overhangs require support for struts above a certain threshold of length/angle, and the removal of supports could be cumbersome. However, the feedstock is safer and easier to handle, and does not require additional post-processing. Furthermore, in special cases, such as in zero gravity manufacturing, FFF is a proven technology [20]. Given the shortcomings of FFF and the availability of alternate AM methods, just a small number of experimental studies have been carried out using FFF.

Other researchers have reported the manufacturing of miniature polymer lattice structures, but information regarding manufacturability or manufacturing restrictions has been sparse. Al Rifaie et al. [21] studied the compression behavior of 3D printed polymer lattice structures. Four variants of the cubic cell (cell size 5 mm and strut thickness 1 mm) were manufactured and tested. Support material was used in some cases, depending on complexity, which was removed in post-processing using a heated chemical bath. Karamooz Ravari et al. [22] fabricated and tested the mechanical properties of a polymer lattice structure. The unit cell was a body-centered cube, with a nominal diameter of struts of 1.5 mm. Some benchmarks were carried out to assess the capabilities of the available AM machine, but results were not reported. More recently, Rossiter et al. [23] evaluated the compressive behavior of truncated octahedron lattice structure (cell size 12–16 mm) manufactured using nylon through the process of FFF. Gautam et al. [24] evaluated the compressive performance of Kagome truss unit cells of acrylonitrile butadiene styrene (ABS) of 35 mm height fabricated by FFF, considering the effect of build orientation and surface quality on its compressive response and energy absorption capacity. Habib et al. [25] compared the performance of octagonal lattices (cell size 10 mm) built by multi-jet fusion (MJF) and FFF, reporting that the MJF fabricated samples had better properties than the FFF samples. Manufacturing of polymer lattice structures by other AM methods has also been reported, including stereolithography [26,27], multi-jet fusion [28], and laser sintering [29,30], but little information is reported regarding manufacturability. Stereolithography and LS are capable of producing small features (strut/wall thickness 0.4–0.6 mm) while values reported for multi-jet fusion are larger (cell size 10 mm; strut diameter > 1.13 mm). Cell forms were limited, and overhang angles were constrained.

An extended summary of research carried out, such as experimental tests and numerical modeling of lattice structures fabricated by AM processes was presented by Dong et al. [25]. Nevertheless, information regarding the capability of FFF to produce miniature cellular structures—or the influence of printing parameters on their quality—is still unresolved. Hence, the limits of the FFF process for building miniature lattice structures of polymeric materials must be explored. The greatest challenge is achieving the quality of the lattice structure, which includes dimensional accuracy, surface quality, residual stresses, material microstructure, trace impurities, and hence variability in material and structural performance.

Previous research has explored the overall capabilities and limitations of various AM methods to produce typical geometrical features. Stratasys [31], offers general design guidelines for material extrusion 3D printing, addressing some limitations such as minimum wall thickness and hole size, as well as the effect of contraction on accuracy.

Adam and Zimmer [32] tested the capabilities of FFF by defining standard features that included elementary geometrical shapes (e.g., cylinders), joints of basic elements, and arrangements of many basic elements, which led to the formulation of general design rules developed for selective laser sintering (SLS), laser melting and FFF.

Schäfer [33] analyzed the restrictions of FFF, which included minimum radii of curvature, wall thickness, hole size, and maximum overhang, among others. Additionally, the influence of layer thickness and printing orientation on precision and surface quality were described. Schäfer proposed FFF design guidelines that considered the restrictions of the process. Considering the typical features of lattice structures, the most relevant guidelines are summarized in Table 1. Although these guidelines were proposed considering the design of large parts, whether they are also applicable to miniature lattice structures is still unclear.

In the present work, we explore the manufacturing of 3D miniature polymer lattice structures using fused filament fabrication, specifically for lattices with cell size and strut diameter close to the smallest dimension possible.

## 2. Materials and Methods

**Figure 1:**
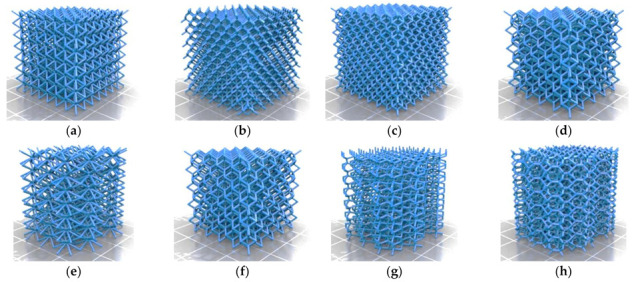
Benchmark of eight lattice structures proposed by Zhou [18]: (**a**) cube vertex centroid; (**b**) cubic diamond; (**c**) cubic fluorid; (**d**) tet vertex centroid; (**e**) hex prism vertex; (**f**) tet oct vertex centroid; (**g**) hex prism diamond; (**h**) hex prism laves phase. Images licensed under the Creative Commons License [19].

A benchmark of eight lattice structures proposed by Zhou [18] was evaluated using the FFF design guidelines (Table 1). The influence of geometry (strut thickness and orientation, cell size, scale factor) and FFF parameters (layer thickness, material) were explored. Open cellular materials are advantageous because they allow visual inspection of the inner lattice structure. Figure 1 shows the digital models (STL files) of the eight lattice structures proposed by Zhou [18]. The lattice structures were designed using the software nTopology [34]. A detailed analysis of the geometric features of the eight lattice structures (dimensions, cell size, strut diameter, strut orientation) is presented in Table 1.

### 2.1. Specimen Manufacturing

The first set of printing tests were run using unbranded PLA as feedstock material and an Ender 3 desktop printer (Creality, Shenzhen, China). There is no information available about chemical composition or other technical specifications, so compression tests of solid samples were performed under standard ASTM D695 [35] to determine its basic mechanical properties.

Additional printing tests were made in a second desktop printer, an Up mini 2 (Tiertime, Beijing, China). White ABS (Tiertime) was used as feedstock material. Slicing software Cura 4.0 (Ultimaker, Utrecht, Netherlands) (for the Ender 3) and Up Studio v2.6 (Tiertime) (for the Up mini 2) were used to process the STL files.

No support was used during the manufacturing process, as the goal was to identify self-supporting lattice structures. Mechanical properties of PLA and ABS feedstock materials are presented in Table 2. Table 3 presents a comparison of both printers and the manufacturing parameters.

Different strategies were tested to improve adhesion and reduce damage when removing the structure from the printer bed, as the fracture of struts was common. The use of brim and raft offered adequate adhesion, but removal from the bed occasionally damaged adjacent struts. The use of masking tape applied to the printer bed covered with a layer of adhesive on its reverse to improve adhesion was favorable.

### 2.2. Compression Tests

A WDW-200E (TIME Group Inc., Beijing, China) computerized electronic universal testing machine with flat plates (diameter 200 mm) was used to carry out the compression tests. Specimens were tested in the same orientation of printing. The preparation of the specimens and the compressive tests were carried out according to the standard test method for compressive properties of rigid cellular plastics, ASTM D1621 [38]. A constant speed of 2.3 mm/min was used during the compression tests. No lubricant was used in the contact surfaces between specimens and plates. Compression tests were carried out until the samples were compressed to about 50% strain, as the densification phase was beyond the scope of this study. For error estimation, two specimens of every configuration were manufactured and tested under uniaxial compression, totaling 32 samples/experiments.

Compressive stress (i.e., plateau stress) and energy absorption capacity for all samples were determined from the experimental data. The plateau stress is defined as the arithmetical mean of stresses at 20% and 40%, according to the standard for mechanical testing of cellular metals ISO 13,314 [39]. The energy absorption capacity quantified was measured as the area under the stress-strain curve up to a value of 50% nominal strain.

## 3. Results

### 3.1. Manufacturability Assessment Based on FFF Design Guidelines

Table 4 presents a summary of the structures to be tested and their characteristics. Parameters exceeding the restrictions of Table 1 were marked with an asterisk. The right column assesses the feasibility, specifying which guidelines were ignored.

### 3.2. Printing Tests

**Figure 2:**
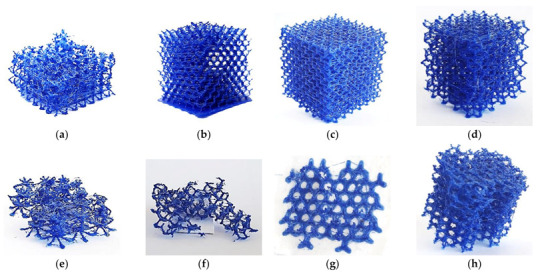
3D printed lattice structures (scale 1:1, Ender 3, PLA): (**a**) cube vertex centroid; (**b**) cubic diamond; (**c**) cubic fluorid; (**d**) tet vertex centroid; (**e**) hex prism vertex; (**f**) tet oct vertex centroid; (**g**) hex prism diamond; (**h**) hex prism laves phase. Dimensions of specimens are reported in Table 4.

The eight lattice structures (Figure 1) were printed on a 1:1 scale using PLA. Tests for lattice structures CD, CF, TV and HPL were partially successful, but structures CVC, HPV, TOV and HPD could not be printed (Figure 2). The overall accuracy and quality of printed structures were poor: struts were missing and both the shape and thickness of struts were inconsistent. Additionally, stringing inside cells and lumps of filament were visible in some cells. Nevertheless, the printed lattice structures were capable of enduring moderate compressive loads, even if struts were fragile or incomplete. These results were consistent with the guidelines summarized in Table 1.

Although the first layers of the CVC lattice could be printed (Figure 1a), the structure became too unstable after the second row of cells to support new layers: extruded filament could not secure adhesion to the previous layers, and the printing failed. Similarly, the first half row of cells in the HPV structure was printed satisfactorily (Figure 1e), even if the overhang of struts exceeds the guidelines (Table 4). However, after the first row of nodes, not a single strut was completed. While the overhang of struts does not change in the second row, the long struts lacked the stability to support new layers, and the extruded filament did not stick to the cantilever struts.

Regarding TOV and HPD lattices (Figure 1f,g), the printer was unable to generate vertical struts, a feature that violated rules 10 and 11 (Table 1). The thin vertical struts could not deposit more than a couple of layers, as the strut was too unstable to secure the deposition of new layers.

**Figure 3:**
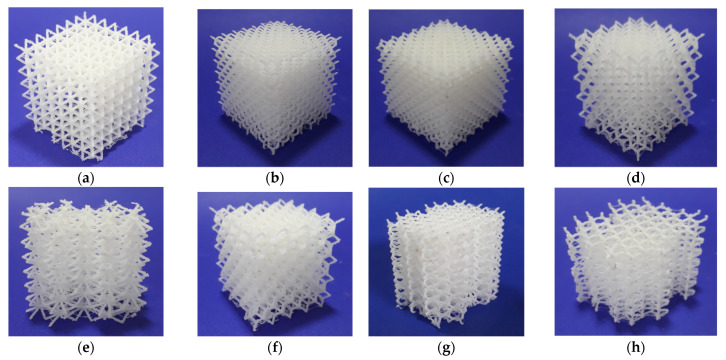
3D printed lattice structures (scale 1:1, Up mini 2, ABS): (**a**) cube vertex centroid; (**b**) cubic diamond; (**c**) cubic fluorid; (**d**) tet vertex centroid; (**e**) hex prism vertex; (**f**) tet oct vertex centroid; (**g**) hex prism diamond; (**h**) hex prism laves phase. Dimensions of specimens are reported in Table 4.

The eight lattice structures printed using ABS as feedstock material and the alternative printer (Up mini 2) are presented in Figure 3. Tests were successful for all lattices. The overall accuracy and quality of printed structures were improved, although minor irregularities, stringing and stair-stepping were noticeable. The printed lattice structures were much resistant than those obtained using PLA. The results of the printing tests for both PLA and ABS are summarized in Table 4.

It was remarkable that the ABS lattice structures could be printed, even when the established design guidelines predicted negative outcomes (Table 5). Hence, further analysis is required to understand the influence of FFF restrictions on the manufacturing of lattice structures.

**Figure 4:**
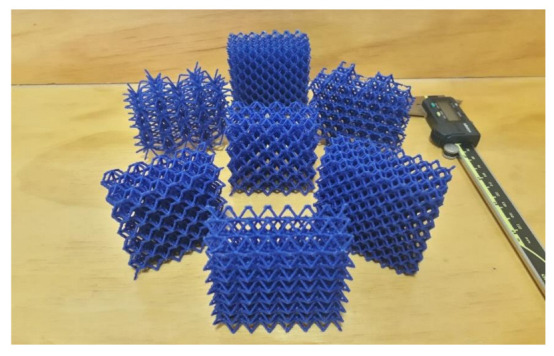
3D printed lattice structures (scale 2:1, Ender 3, PLA). Lattice structure HPV could not be printed.

A third round of printing tests using larger models (scale 2:1) was carried out using PLA and the Ender 3 printer. Height of these specimens was limited to 30 mm, enough to assess their manufacturability. All lattice structures except HPV were printed satisfactorily (Figure 4). Table 6 summarizes the results for the printing tests of the eight lattice structures on a 2:1 scale.

### 3.3. Compression Tests

**Figure 5:**
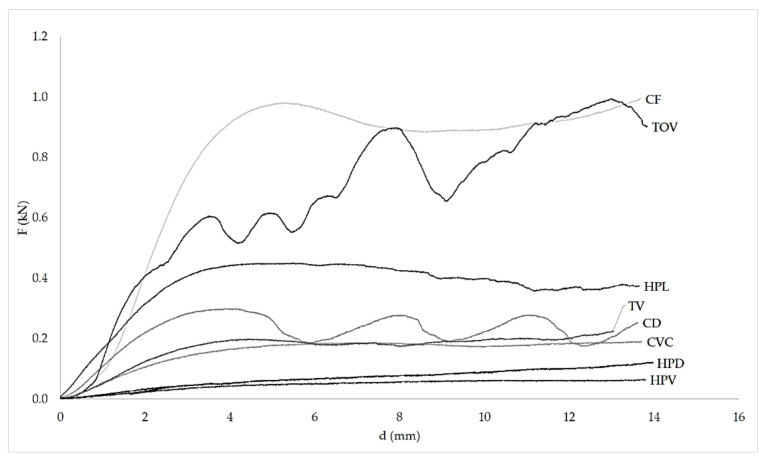
Force-displacement diagram for the eight ABS lattice structures. Please improve the quality of Figure 5, Figure 6, Figure 7 and Figure 8 and try to keep the consistency in the plotting figures.

Figure 5 shows the force-displacement curve for the eight ABS lattice structures that were successfully printed. Compression tests were carried out for PLA lattice structures (scale 1:1), but their strength was significantly lower. The region of densification that commonly follows the plateau is not depicted in Figure 5, as the compression tests were ended at 50% nominal strain, before reaching densification strain.

**Figure 6:**
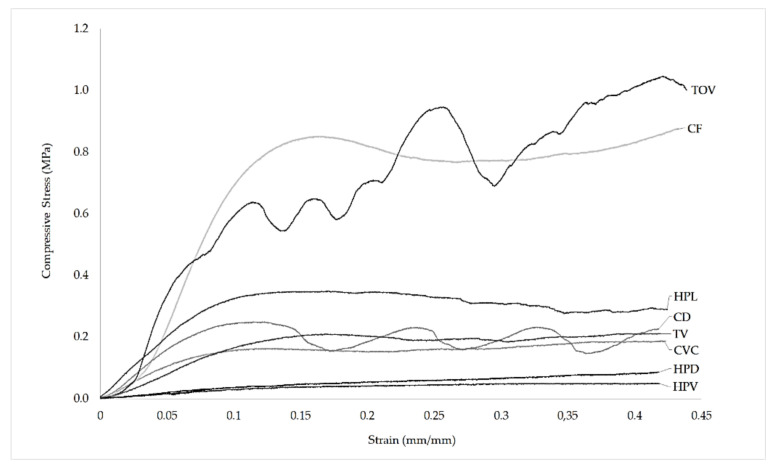
Stress-strain diagram for the eight ABS lattice structures.

Figure 6 shows the compression stress-strain curve for the eight lattices. Stress is calculated using the full cross-section area of the specimens. The stress-strain curves show nearly linear elasticity at low stresses, followed by a stress plateau, a behavior that is typical of bending-dominated lattice structures [40]. TOV and CD, unlike the other lattice structures, showed significant oscillations of plateau stress during testing.

Mean values and standard deviations (SD) for the elastic modulus, plateau stress and energy absorption capacity of the eight ABS lattice configurations are presented in Table 7. The energy absorption capacity quantified was measured as the area under the stress-strain curve up to a value of 40% nominal strain.

**Figure 7:**
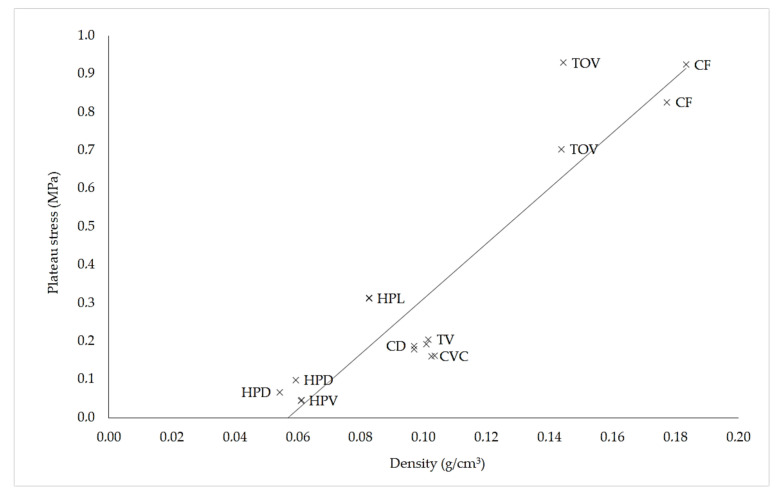
Plateau stress vs. density for the sixteen ABS specimens. Two tests were run for each lattice structure. The line depicts the overall relationship between plateau stress and density.

Figure 7 presents the relationship between plateau stress and density for the sixteen specimens of ABS lattice structures. Although some lattices deviate from the global tendency, the well-known relationship between plateau stress and relative density is evident.

**Figure 8:**
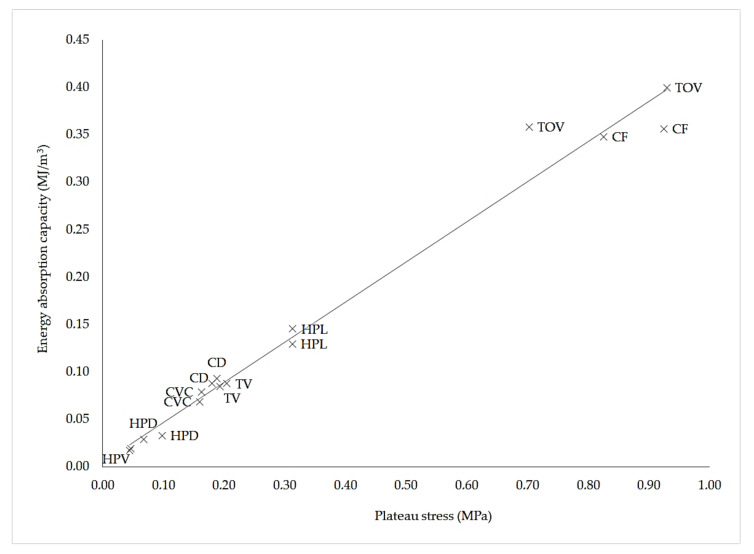
Energy absorption capacity vs. plateau stress for the sixteen ABS specimens. Two tests were run for each lattice structure.

Figure 8 presents the relationship between energy absorption capacity and plateau stress for the sixteen ABS specimens. Although the TOV and CD lattice structures showed strong variations in their plateau stress, the linear relationship between energy absorption capacity and plateau stress is essentially linear.

## 4. Discussion

### 4.1. Influence of Size and Geometry

It was not possible to print miniature lattices CVC, TOV, HPV, and HPD in the Ender 3 printer using PLA filament, on a 1:1 scale. This is consistent with FFF design guidelines proposed in the literature, as strut diameter was too small, and both long vertical struts and excessive overhangs were present. On the other hand, CD, CF, TV, and HPL lattices were printed, although the structures had poor quality and low compressive strength, a consequence of the small strut diameter and large overhangs present in these structures. Two features stood out in the four lattices that could not be printed: long inclined struts (4 mm or longer in CVC and HPV) or vertical struts (TOV and HPD).

**Figure 9:**
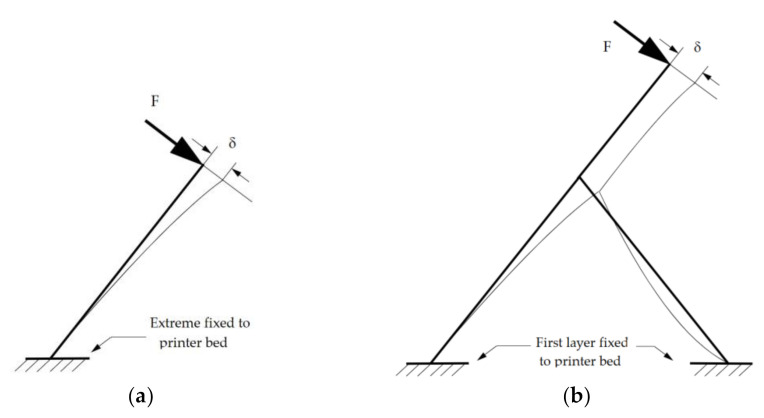
Influence of strut position on its deflection (δ); (**a**) deflection in slender struts fixed directly to the printer bed is determined by strut geometry; (**b**) deflection of struts connected to nodes (upper rows) depends on both strut geometry and stability of the lattice.

Difficulties with long inclined struts arose after the second row of struts (HPV and CVC), which suggests that both strut length and relative position in the lattice influenced the outcome. Long inclined struts in both HPV and CVC behave as cantilever beams during printing, deflecting under the relatively low force exerted when the extruded filament is being deposited. If the strut is fixed to the printing bed, strut support is high and deflection is tolerable. However, as the structure grows taller, strut support becomes more flexible, as both deflection strut and lattice get compounded (Figure 9). Layer deposition cannot find proper support and subsequent layers fail.

Respecting TOV and HPD lattices, thin vertical struts with a length over 0.5 mm could not be printed. Although a few layers could be deposited, subsequent layers could not find proper support and filament failed to adhere to previous layers.

The overall poor results of the first set of printing tests were consistent with previous analysis of dimensional accuracy in FFF; for a nominal diameter in the range 0–3 mm the corresponding tolerance class for FFF is IT14, about ±0.2 mm [41]. As the accuracy was about the same size of strut thickness, deviations were substantial when printing the miniature lattices. The additional material that was not deposited correctly, ended up in other struts or piled up around the nozzle, eventually colliding with other struts, forming lumps or damaging parts of the already printed structure.

**Figure 10:**
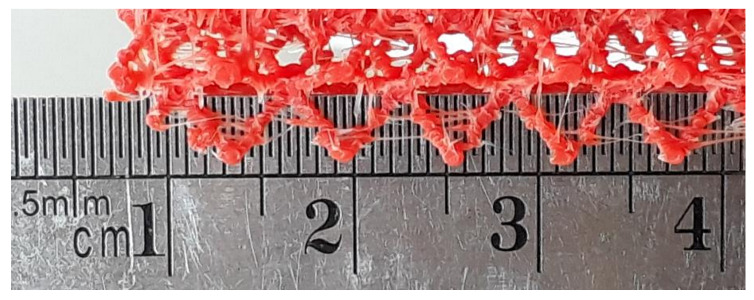
Multiple adjacent struts (lattice structure 1, ABS, Ender 3 scale 1.25:1). Nominal strut thickness 0.9 mm.

In general, filament deposition on thin struts (thickness 0.7–0.9 mm) was challenging. In thin struts (below 0.9 mm thickness) filament is deposited as droplets, each one representing the entire cross-section of the strut (Figure 10). The successful deposition of a droplet depends on the accurate position of the droplets in previous layers, and large errors could compromise successive layers. Furthermore, droplet size and shape change during the fabrication process, according to nozzle trajectory, filament flow and filament-droplet-layer interaction. Hence the droplet-strut interaction is critical and stringing could lead to misprints, as the strings drag part of the extruded filament.

**Figure 11:**
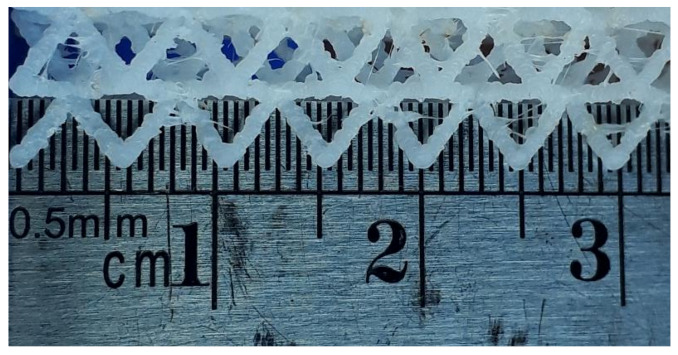
Multiple adjacent struts (lattice structure 1, ABS, Up mini 2, scale 1:1). Nominal strut thickness 0.7 mm. Effective strut thickness 0.89 ± 0.06 mm.

On the other hand, in the second round of tests (Up mini 2, ABS), all eight lattice structures could be fabricated at a 1:1 scale. An analysis of the struts (Figure 11) reveals a higher accuracy in the deposition of filament in the struts manufactured using the Up mini 2 printer.

The stark contrast between the first and second set of printing tests could be traced back to two critical differences: FFF hardware setup [42], and slicing software [43].

While both slicing software were capable of generating the instructions for the 3D printers, intrinsic differences in their operation could affect the dimensional accuracy in AM parts [43]. For instance, nominal thickness in the CVC lattice was 0.7 mm, but the effective strut thickness was around 0.89 mm in ABS printed parts, and 0.75 mm in PLA lattices. However, even if a larger strut diameter does represent an advantage during fabrication, the Ender 3 printer also evidenced shortcomings when printing lattice with a 0.9 mm strut diameter. Figure 10 shows struts printed in the Ender 3 with a nominal thickness of 0.9 mm, with clear signs of poor connection between layers, hinting at restrictions related to the hardware setup of the printer.

**Figure 12:**
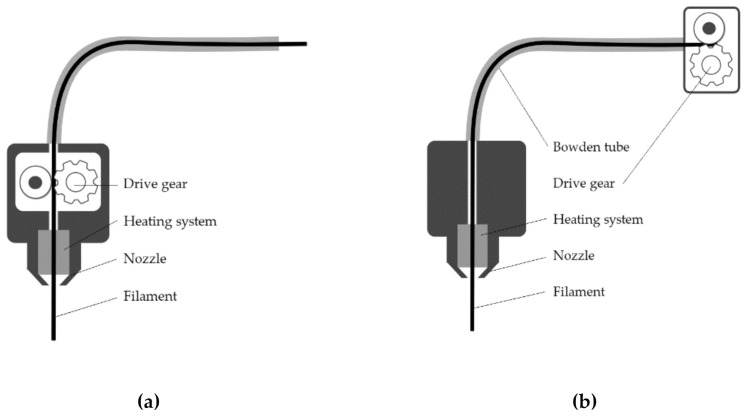
Two types of extruder head configurations: (**a**) a direct drive extruder, with both gear drive and heating system are mounted on the extruder head, and (**b**) Bowden drive, with the drive gear placed outside the extruder head.

**Figure 13:**
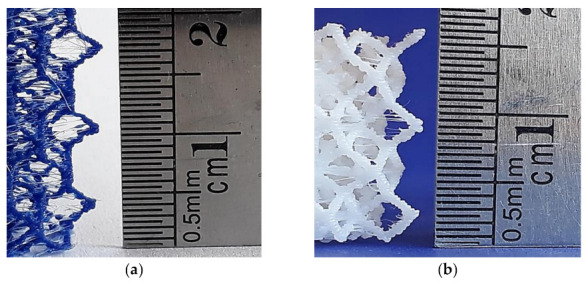
Detail of two TV lattice structures printed using different printers at a 1:1 scale: (**a**) TV lattice printed in Ender 3 (PLA); (**b**) TV lattice printed in Up mini 2 (ABS).

One key difference between both printers—Ender 3 and Up mini 2—that could have influenced the outcome is the extruder type. The Up mini 2 has a direct drive gear, characterized by the location of the drive close to the heating system and nozzle (Figure 12a). Extrusion and retraction commands are more immediate, creating a more stable material deposition control and better resolution [44]. On the other hand, the Ender 3 has a Bowden drive, in which the drive gear is installed outside the extruder head (Figure 12b). Although retraction settings for the Ender 3 were set at high values to prevent unnecessary extrusion while the extruder was traveling between printed sections [44], the result was nevertheless poorly constructed struts in lattices fabricated at a 1:1 scale (Figure 13a). The control of filament flow and deposition in Bowden could be regulated through additional features, like combing and Z-hop [45], but these are not practical in miniature lattices due to the small diameter of struts.

**Figure 14:**
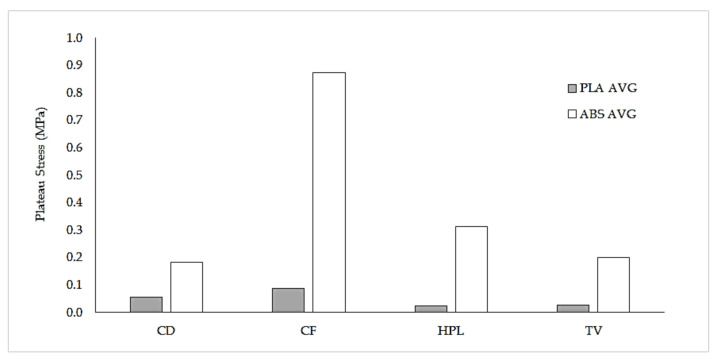
Comparison of plateau stress between PLA and ABS lattices (scale 1:1). PLA specimens were built using the Ender 3 (Bowden drive), while the ABS specimens were built using the Up mini 2 (direct drive).

The effect of the hardware (i.e., type of extruder) is not only evident in the geometrical accuracy of the struts, but also in the mechanical properties of lattices. Figure 14 shows a comparison of plateau stress between specimens printed using the Ender 3 (Bowden drive, PLA), and those fabricated using the Up mini 2 (direct drive, ABS). Although the mechanical properties of both ABS and PLA are quite similar (Table 2), PLA lattices show significantly lower plateau stress values than ABS structures, a consequence of the overall lower accuracy and strength of individual struts: while struts fabricated using the Bowden drive 3 are thin, irregular and brittle (Figure 13a), struts produced by the direct drive are thicker and tougher, and show regular layers (Figure 13b).

In the third round of printing tests (Ender 3, PLA, scale 2:1), in which the strut diameter was 1.4 mm, the stringing inside the cells was practically eliminated and filament deposition in struts was successful. Nevertheless, the fabrication of lattice HPV was not possible, as the printer could build neither vertical struts nor the transitions from vertical to inclined struts. This is consistent with design guidelines (Table 1): vertical struts can only be printed if their height-to-diameter ratio is small; as for inclined struts, it is not possible to print struts with overhang angles over 65° and a length over 2 mm, as FFF cannot secure the support to print large overhangs with such a steep angle.

**Figure 15:**
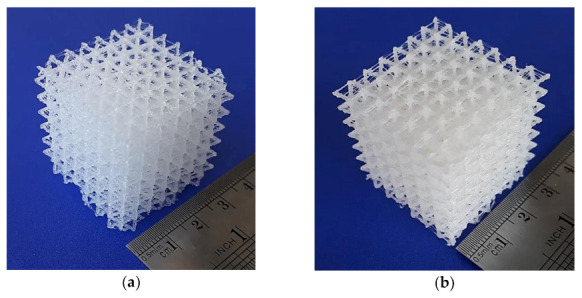
3D printed lattice structures (scale 1:1, Up mini 2): (**a**) CVC printed using PLA; (**b**) CVC printed in PC.

To confirm the influence of the type of extruder, additional printing tests using PLA and polycarbonate (PC) as feedstock materials were carried out in the Up mini 2 printer. CVC lattices (scale 1:1) were successfully printed using both materials, with a quality similar to that of ABS lattices (Figure 15).

### 4.2. Characterization of ABS Lattice Structures

Although the manufacturing of small geometric features (1 mm or less) pushed the FFF to its limits, evidenced in struts with noticeable stair-stepping, the influence of irregularities in struts on the overall strength of the lattice was not critical: all tested samples were capable of sustaining compressive loads without evidence of premature failure.

The largest plateau stress values were obtained for the lattices with the highest density CF and TOV. On the other hand, the lowest values of plateau stress were obtained for structures with the lowest density, lattices HPV and HPD. This outcome is consistent with the relationships proposed in the literature [40,46]. Nevertheless, deviations from the overall tendency were noticeable for the different lattice configurations, suggesting that the type of unit cell might influence the plateau stress. Whether this influence is strictly a consequence of the variation in density or unit cell geometry could not be established, as some variation between specimens of the same lattice structure was noticeable.

One interesting observation during the compression tests was that some lattice structures showed partial elastic recovery (up to 80% of their original height) after the load was removed. Similar behavior has been reported previously for SLA lattices [26], and other materials [47,48]. The structural design of the lattice was decisive to determine the elastic response of the lattice. While in some lattices struts fractured under stress (CD, TOV, HPL) or were deformed permanently (CF), in other configurations a large percentage of struts bent without suffering permanent damage (CVC, HPV, HPD), with the nodes acting as hinges. As the deformed beams pivoted backward on their hinges, the lattice recovered elastically. Maxwell’s stability criterion, might be used to identify which lattices are statically and kinematically determinate (i.e., they are rigid and do not fold up when loaded) [40].

The absorbed energy up to 50% of the nominal strain of lattices CF and TOV was 0.3521 and 0.3790 MJ/m^3^, respectively. Values of energy absorption capacity (Table 7) were similar to those reported for truncated octahedron lattices built in nylon with large cells (>12 mm) (0.035–1.069 MJ/m^3^) [23] and superior to those of large ABS Kagome unit cells (0.1–0.14 MJ/m^3^, cell size 35 mm) [24] and SLA octet miniature lattices of similar density (0.005–0.025 MJ/m^3^) [26].

## 5. Conclusions

Miniature 3D open cell lattice structures could be manufactured by FFF without using support material, using complex unit cell geometries not limited to the classic cubic-type cell arrangements. The 3D printed miniature lattice structures were capable of supporting compressive loads, even when overall quality and accuracy were not optimal. While thickness and orientation of the struts were identified as important parameters to assess the manufacturability of miniature lattice structures, hardware setup is considered a critical factor.

In conventional Bowden extruder printers, general design guidelines should be observed: strut diameter should not be smaller than 1 mm, and there are limits of length for both vertical and inclined struts depending on overhang angle: the steeper the angle, the shorter the strut. In vertical struts, the length-to-diameter ratio should not exceed the recommended value. On the other hand, although manufacturing restrictions could be circumvented using a direct drive extruder, there are hard limits to the FFF process.

Further research is required to characterize the properties of the eight lattices and the influence of geometric parameters on their mechanical behavior. There is potential for the development of cellular metals based on polymer lattice structures, as the printed structures could be used as patterns for investment casting or electro-deposition techniques [49,50]; FFF could also directly produce cellular metals through the material extrusion additive manufacturing (FFF), debinding, and sintering route [51]. Even if printing errors cannot be completely avoided when 3D printing lattice structures, the process could offer some advantages when compared to other available methods.

The validity of the results could be expanded to other feedstock materials, such as polycarbonate and possibly polyetheretherketone (PEEK). PEEK, a semi-crystalline thermoplastic with excellent biocompatibility and mechanical properties that could be printed using FFF processes [52,53,54], could be of special interest, as it would enable its use in applications such as orthopedic implants.

Further study of the hardware setup of printers, for instance, the type of extruder (direct vs. Bowden) or the use of smaller nozzle diameters (0.2 mm) when printing miniature geometric features is recommended, as the literature dealing with both topics is scarce.

Finally, accuracy assessments of the printed lattices—although not widely available—could be used in the future to compare the performance of different AM processes. For instance, 3D tomography could be used to reconstruct the geometry of printed lattice and generate a digital image of the real structure, which can be compared with the original STL model [14,55].

## Figures and Tables

**Table 1:** Relevant FFF design guidelines for lattice structures, after Schäfer [33].

	Features	Restriction	Comment
1	Wall thicknesses	>1.5 mm	All contours and orientations
2	Floor thicknesses	>1 mm	For layer thickness = 0.2 mm
3	Hole diameters	>2 mm	Form tolerance
4	Cylinder diameter	>2 mm	Form tolerance
5	Section	>5 mm × 5 mm	For individual structures
6	Radius	>1 mm	Form tolerance
7	Grooves or channels	>0.3 mm	
8	Wall thicknesses	>1 mm	
9	Wall length	>5 mm	For individual structures
10	Vertical cylinder diameter	>3 mm	
11	Vertical cylinder height/cross-section ratio	>1.5	
12	Overhang angles	>45°	For surface quality

**Table 2:** Mechanical properties of PLA and ABS.

Feedstock Material	PLA	ABS [36,37]
Density (g/cm^3^)	1.167	1.195
Elastic modulus (MPa)	1431	2180
Compressive strength (MPa)	60	64

**Table 3:** Configuration of the printers and printing parameters.

Printing Parameters	PLA	ABS
Extruder drive	Bowden	Direct
X-movement	Extruder	Extruder
Y-movement	Bed	Bed
Z-movement	Extruder	Bed
Printing volume (cm)	220 × 220 × 250	120 × 120 × 120
Nozzle diameter (mm)	0.4	0.4
Extruder temperature (°C)	200	270
Bed temperature (°C)	50	90
Layer height (mm)	0.16	0.15
Extrusion width(mm)	0.2	0.3
Printing speed (mm/s)	60	undisclosed *
Support	No	No
Surface adhesion	Raft	Raft

* not reported by printer/software.

**Table 4:** Geometric features of tested unit cells and lattice structures, and contravened FFF guidelines.

		Structure			Struts			Cell Size (mm)	Contravened Guideline
		Height (mm)	Width (mm)	Length (mm)	Diameter (mm)	Length (mm)	Overhang Angle (°)		
CVC	Cube vertex centroid	31	31	31	0.70 *	4	45	5	4
CD	Cubic diamond	34	34	34	0.70 *	2.5	65 *	3	4
CF	Cubic fluorid	33	33	33	0.70 *	2.5	65 *	4	4, 12
TV	Tet vertex centroid	30	30	30	0.70 *	3	55 *	5.5	4, 12
HPV	Hex prism vertex	34	34	34	0.70 *	5	60 *	4.5/6.5	4, 12
TOV	Tet oct vertex centroid	31	31	31	0.70 *	3/3/4.5	0/55 */90 ^+^	4.5/6.5	4, 10, 11, 12
HPD	Hex prism diamond	34	34	39	0.70 *	3/2	65 */90 ^+^	3/5	4, 10, 11, 12
HPL	Hex prism laves phase	34	34	39	0.70 *	3/2	0/60 *	4.5/5.5	4, 12

* Exceeds critical design value according to Table 1. + Exceeds critical design value, but bridging is possible.

**Table 5:** Results of 3D printing tests of lattice structures for both PLA and ABS, scale 1:1.

			Results		Contravened Guideline
		Feasible?	PLA	ABS	
CVC	Cube vertex centroid	No	VP	A	4
CD	Cubic diamond	No	P	A	4, 10
CF	Cubic fluorid	No	P	A	4, 12
TV	Tet vertex centroid	No	P	A	4, 12
HPV	Hex prism vert	No	F	A	4, 12
TOV	Tet oct vertex centroid	No	F	A	4, 10, 12
HPD	Hex prism diam	No	F	A	4, 10, 12
HPL	Hex prism laves phase	No	VP	A	4, 12

A: Acceptable; F: Failed; P: Poor; VP: Very Poor.

**Table 6:** Results of FFF printing tests of larger lattice structures for PLA, scale 2:1.

	Structure	Outcome	Comments
CVC	Cube vertex centroid	A	minor stringing.
CD	Cubic diamond	A	stair-stepping.
CF	Cubic fluorid	A	stair-stepping.
TV	Tet vertex centroid	A	stair-stepping.
HPV	Hex prism vert	A	stair-stepping.
TOV	Tet oct vertex centroid	A	stair-stepping.
HPD	Hex prism diam	F	
HPL	Hex prism laves phase	A	stair-stepping.

A: Acceptable; F: Failed; P: Poor; VP: Very poor.

**Table 7:** Mean values and standard deviation for ABS lattice structures.

ID	Density (g/cm^3^)		Plateau Stress (MPa)		Energy Capacity (MJ/m^3^)	
	Mean	SD	Mean	SD	Mean	SD
CVC	0.1030	7.07 ×10^−4^	0.1617	1.77 ×10^−3^	0.0738	7.33 ×10^−3^
CD	0.0971	7.07 ×10^−5^	0.1840	5.66 ×10^−3^	0.0908	3.81 ×10^−3^
CF	0.1805	4.31 ×10^−3^	0.8750	7.00 ×10^−2^	0.3521	5.92 ×10^−3^
TV	0.1013	3.54 ×10^−4^	0.1988	8.13 ×10^−3^	0.0865	1.96 ×10^−3^
HPV	0.0612	2.12 ×10^−4^	0.0458	1.06 ×10^−3^	0.0182	1.36 ×10^−3^
TOV	0.1442	4.24 ×10^−4^	0.8160	1.61 ×10^−1^	0.3790	2.93 ×10^−2^
TPD	0.0569	3.54 ×10^−3^	0.0830	2.19 ×10^−2^	0.0313	2.69 ×10^−3^
HPL	0.0828	7.07 ×10^−5^	0.3130	7.07 ×10^−4^	0.1377	1.12 ×10^−2^

## Data Availability

Data available on request.
